# Clozapine-Treatment-Resistant Schizophrenia Successfully Managed with Brexpiprazole Combination Therapy: Two Case Reports

**DOI:** 10.1192/j.eurpsy.2022.903

**Published:** 2022-09-01

**Authors:** L. Orsolini, S. Tempia Valenta, S. Bellagamba, V. Salvi, U. Volpe

**Affiliations:** Unit of Clinical Psychiatry, Polytechnic University of Marche, Department Of Neurosciences/dimsc, Ancona, Italy

**Keywords:** schizophrénia, Brexpiprazole, Clozapine-Treatment-Resistant, Combination Therapy

## Abstract

**Introduction:**

Clozapine has proven to have a unique efficacy on treatment-resistant schizophrenia (TRS). Nevertheless, studies show that 47%-63% of clozapine-treated patients may fail to respond after around 12-years of treatment (CRS). Several augmentation strategies have been proven to be effective in CRS.

**Objectives:**

Hereby, we present two clinical cases of CRS successfully managed with brexpiprazole augmentation.

**Methods:**

A 48-year-old man without comorbid substance use, treated with clozapine-brexpiprazole augmentation, and a 20-year-old man with comorbid substance use, treated with clozapine-brexpiprazole combination and subsequently with twice-injection aripiprazole (TIA). They were administered with the following assessments at t0, t1-3 (first month), t4-8 (monthly until 6-month follow-up): CGI, BPRS, PANSS, CDSS, Craving VAS, BARS, BIS-11, HRS-A, MADRS, YMRS, AIMS.

**Results:**

At 1-month follow-up, both patients showed a considerable improvement (respectively 75% and 55.9% reduction of PANSS total score). At 6-month follow-up, reached only with the first patient, we noticed a further improvement (an overall 37.5% reduction of PANSS total score from the baseline).

**Conclusions:**

The present work is the first report describing combination treatment strategies with clozapine and brexpiprazole which appear to give promising results.

**Disclosure:**

No significant relationships.

